# Beyond Pain Intensity: Central Sensitization and Psychosocial Symptom Burden in Women with Rheumatoid Arthritis

**DOI:** 10.3390/medicina62091780

**Published:** 2026-09-16

**Authors:** Maja Vučković, Dragana Kožul, Tamara Popović, Ivan Soldatović, Sandra Trivunović, Tatjana Nožica Radulović, Daria Ćupurdija, Snežana Tomašević Todorović

**Affiliations:** 1 Institute for Physical Medicine, Rehabilitation and Orthopedic Surgery “Dr Miroslav Zotović”, 78000 Banja Luka, Republic of Srpska, Bosnia and Herzegovina; kozuldragana1@gmail.com (D.K.); tamaralukac@yahoo.com (T.P.); sandra.tepic@yahoo.com (S.T.); tatjananozica@gmail.com (T.N.R.); 2 Faculty of Medicine, University of Banja Luka, 78000 Banja Luka, Republic of Srpska, Bosnia and Herzegovina; 3 Institute for Medical Statistics and Informatics, Faculty of Medicine, University of Belgrade, 11000 Belgrade, Serbia; soldatovic.ivan@gmail.com; 4 School of Medicine, University of Mostar, 88000 Mostar, Federation of Bosnia and Herzegovina, Bosnia and Herzegovina; dariaostojic@gmail.com; 5 Clinic of Physical Medicine and Rehabilitation, University Hospital Mostar, 88000 Mostar, Federation of Bosnia and Herzegovina, Bosnia and Herzegovina; 6 Clinic for Physical Medicine and Rehabilitation, Clinical Center of Vojvodina, 21000 Novi Sad, Serbia; drtomasevic@gmail.com; 7 Faculty of Medicine Novi Sad, University Clinical Center of Vojvodina, 21000 Novi Sad, Serbia

**Keywords:** arthritis, rheumatoid, central sensitization, chronic pain, depression, fatigue, rehabilitation

## Abstract

*Background and Objectives*: Persistent pain in rheumatoid arthritis (RA) may reflect mechanisms not fully captured by systemic inflammatory markers. This study estimated the frequency of elevated Central Sensitization Inventory (CSI) scores in women with RA and examined associations with pain, fatigue, depressive symptoms, function, inflammatory markers, and disease activity. *Materials and Methods*: This prospective single-center observational cohort included 97 female inpatients who completed a standardized three-week rehabilitation program. The CSI was used as a symptom-based screening instrument. Pain, function, fatigue, and depressive symptoms were assessed with the Visual Analog Scale (VAS), Health Assessment Questionnaire (HAQ), FACIT-Fatigue scale, and Beck Depression Inventory-II (BDI-II), respectively. *Results*: Fifty-seven participants (58.8%) had CSI scores ≥ 40. Higher CSI scores were associated with greater pain, more depressive symptoms, more fatigue, and higher DAS28-CRP scores, but not with ESR, CRP, IL-6, or disease duration. In adjusted baseline models, the association with VAS pain was statistically significant but modest (unstandardized beta = 0.024 per CSI point; standardized beta = 0.273). Across the three-week follow-up, VAS pain decreased by 2.53 points; mean FACIT-Fatigue and HAQ changes were smaller than commonly cited clinically important thresholds, and CSI changed only modestly. In adjusted multivariable models, higher CSI scores remained independently associated with VAS pain, FACIT-Fatigue, and BDI-II scores, whereas no significant association was found with HAQ. CSI was not associated with CRP, IL-6, or disease duration. *Conclusions*: In this cohort of women with RA, elevated CSI scores identified a greater self-reported symptom burden and were independently associated with pain, fatigue, and depressive symptoms after adjustment, though the pain association remained modest in magnitude. No meaningful association was found with functional disability. The uncontrolled, single-arm design and restricted inflammatory-marker range preclude causal conclusions or proof that symptoms were independent of inflammation.

## 1. Introduction

Rheumatoid arthritis (RA) is a chronic, systemic, immune-mediated autoimmune disease. Clinically, it manifests as persistent inflammatory arthritis accompanied by symmetric polyarticular pain and swelling, most commonly of the small joints of the hands and feet, as well as extra-articular manifestations [1]. Its etiopathogenesis involves a complex interaction between genetic predisposition and environmental factors [2]. In clinical practice, RA is diagnosed on the basis of symptoms and signs of active joint inflammation, as well as biomarkers such as autoantibodies and radiographic changes that may demonstrate joint inflammation and/or damage. Based on the presence or absence of elevated serum autoantibody levels (rheumatoid factor (RF) and/or anti-citrullinated protein antibodies—ACPA), RA is classified as seropositive or seronegative [3]. In addition, established classification criteria for RA include the 1987 American College of Rheumatology (ACR) criteria and the 2010 ACR/European Alliance of Associations for Rheumatology (EULAR) criteria [4]. The disease represents a major health problem because of its chronic course and numerous consequences that substantially impair patients’ quality of life.

Pain is one of the most dominant symptoms of this disease and is often the main reason why patients seek medical attention. According to the definition of the International Association for the Study of Pain, pain is “an unpleasant sensory and emotional experience associated with actual or potential tissue damage, or described in terms of such damage” [5]. Although pain in RA has traditionally been considered a consequence of inflammatory processes in the synovium and peripheral tissues, numerous studies indicate that pain intensity in a subset of patients does not always correlate with objective indicators of inflammatory disease activity [6,7,8]. Inflammation is driven by proinflammatory cytokines such as tumor necrosis factor (TNF)-alpha, interleukin (IL)-1, and IL-6, resulting in synovitis or structural joint damage [9]. However, in a substantial number of patients, pain persists despite adequate control of inflammation and the use of modern antirheumatic therapy. This indicates the presence of additional mechanisms involved in pain modulation [10,11].

In the pathogenesis of enhanced pain perception in rheumatoid arthritis, central sensitization stands out as one of the key mechanisms that condition the amplification of nociceptive signals at the spinal cord level. The neurobiological basis of this process consists of reduced levels of serotonin and noradrenaline, which impair descending pain inhibition, and increased glutamatergic activity in the insular cortex, which lowers the pain threshold [12,13,14].

Pain processing differs between women and men through interacting biological and psychosocial pathways; therefore, a women-only cohort cannot be assumed to represent men with RA [15].

In addition to pain, depression is one of the most common accompanying symptoms of RA and is often accompanied by pronounced fatigue [16]. In a significant proportion of patients, depression further amplifies pain perception. Fatigue and depression in RA are considered to result from the complex effects of psychosocial and neurobiological mechanisms and central sensitization, and they cannot be explained by active disease alone [17].

The relationship between CSI symptom burden, fatigue, depressive symptoms, and objective measures of disease activity remains incompletely understood. Therefore, this study aimed to estimate the frequency of elevated CSI scores in women with RA; examine unadjusted associations with pain, fatigue, depressive symptoms, function, disease activity, and inflammatory markers; and evaluate whether baseline CSI scores were associated with VAS pain, HAQ function, FACIT-Fatigue, and BDI-II scores after adjustment for selected inflammatory measures.

## 2. Materials and Methods

### 2.1. Study Design and Participants

The study was designed as a prospective single-center observational cohort with a baseline cross-sectional analysis and a single-arm before–after component. One hundred women with RA were enrolled. Three individuals discontinued rehabilitation after enrolment because of deterioration in their general condition; these cases represent attrition rather than exclusion at baseline. The final analysis included the 97 participants who completed the 21-day inpatient rehabilitation program at the Institute for Physical Medicine, Rehabilitation and Orthopedic Surgery “Dr Miroslav Zotović”, Banja Luka. Patients who withdrew from the study did so due to clinical worsening during the rehabilitation program, resulting in discontinuation before completion of the three-week protocol. These patients did not differ from the remaining participants with respect to baseline demographic or clinical characteristics, and their worsening could not have been anticipated at enrollment.

Inclusion criteria comprised female patients aged 18 to 70 years who had previously provided informed consent to participate in the study. Exclusion criteria were diabetes mellitus, history of cerebrovascular insult, malignancy, alcohol dependence, pregnancy, peripheral nervous system disorders, surgical interventions during the previous six months, and use of antidepressant therapy or pregabalin/gabapentin. The protocol enrolled only women; accordingly, every population-level inference in this manuscript is restricted to women with RA.

None of the participants were receiving psychological therapy during the study period. Baseline depressive symptom severity was assessed with the BDI-II. Although the BDI-II was repeated at discharge (day 21), the present manuscript uses BDI-II only in baseline cross-sectional analyses and does not report a before–after BDI-II change. No psychological intervention was administered.

Before the study began, ethical approval was obtained from the Ethics Committee of the Institute for Physical Medicine, Rehabilitation and Orthopedic Surgery “Dr Miroslav Zotović”, Banja Luka (Chairperson: Tatjana Bućma; Protocol No.: 116-01-20348-2/22; date of approval: 7 October 2022). The study was conducted in accordance with the 1964 Declaration of Helsinki and its later amendments.

Before inclusion in the study, all participants were thoroughly informed about the aims, methods, and conduct of the research, after which they provided written informed consent to participate in the study and to the publication of their data. Participation was voluntary, with the possibility of withdrawal at any time without consequences for further treatment and rehabilitation.

Data were collected from medical history, physical examination, available medical documentation, biosociodemographic questionnaires, and specific questionnaires used in rheumatoid arthritis.

### 2.2. Data Collection and Variables Monitored

Demographic and clinical variables included age, height, body weight, body mass index (BMI), smoking status, disease duration, systemic comorbidities, and treatment with conventional synthetic or biologic disease-modifying antirheumatic drugs (DMARDs). Pain intensity was assessed on a 0–10 Visual Analog Scale (VAS), with higher scores indicating greater pain [18]. Functional status was assessed with the Health Assessment Questionnaire (HAQ; range 0–3), with higher scores indicating greater impairment [19].

Central sensitization-related symptoms were assessed with the 25-item Central Sensitization Inventory (CSI; range 0–100). Each item is scored from 0 to 4, and higher totals indicate greater symptom burden. A score ≥ 40 was classified as an elevated or screening-positive CSI result [20]. Because the CSI is a self-report screening instrument, this threshold was not treated as a definitive diagnosis of central sensitization [21]. Fatigue was assessed with the FACIT-Fatigue scale, on which higher scores indicate less fatigue [22], and depressive symptom severity was assessed with the 21-item BDI-II, a self-report questionnaire in which each item is scored from 0 to 3, yielding a total score ranging from 0 to 63. Scores were classified as minimal (0–13), mild (14–19), moderate (20–28), or severe (29–63), with higher scores indicating greater depressive symptom severity [23,24].

### 2.3. Assessment of Disease Activity and Inflammatory Parameters

Disease activity was assessed using DAS28-ESR and DAS28-CRP. Systemic inflammatory measures were erythrocyte sedimentation rate (ESR, mm/h), C-reactive protein (CRP, mg/L), and serum interleukin-6 (IL-6, pg/mL). Blood samples were collected by trained laboratory technicians. Serum IL-6 was measured by electrochemiluminescence immunoassay on the Cobas e601 analyzer (Roche Diagnostics GmbH, Mannheim, Germany) by a specialist in clinical biochemistry.

DAS28 incorporated the 28 tender-joint count (TJC28), 28 swollen-joint count (SJC28), patient global assessment (PGA; 0–100), and an acute-phase reactant. DAS28-ESR was calculated as 0.56 × sqrt(TJC28) plus 0.28 × sqrt(SJC28) plus 0.70 × ln(ESR) plus 0.014 × PGA. DAS28-CRP was calculated as 0.56 × sqrt(TJC28) plus 0.28 × sqrt(SJC28) plus 0.36 × ln(CRP plus 1) plus 0.014 × PGA plus 0.96. Scores below 2.6 indicate remission, 2.6 to below 3.2 indicate low activity, 3.2 to 5.1 indicate moderate activity, and above 5.1 indicate high activity [25]. Tender joint count (TJC28) and swollen joint count (SJC28) were assessed by a rheumatologist. Radiographic staging was performed using standard (conventional) radiography and scored by a rheumatologist according to modified Steinbrocker criteria. RF and ACPA had been assessed previously as part of routine clinical care, but complete results were not available for all participants. Serostatus was therefore not systematically analyzed or reported in the present study.

### 2.4. Rehabilitation Protocol

During medical rehabilitation, all patients received a standardized physical therapy protocol that included an individualized kinesitherapy program and functional occupational therapy, conducted six days per week for three weeks. Analgesic electrotherapy procedures included pulsed electromagnetic field therapy using a magnetic mat at a frequency of 19 Hz and 10 mT for 30 min, for a total of 10–15 treatments, and galvanic baths applied at a water temperature of approximately 35 °C with a current intensity of 10–20 mA for 20 min, for a total of 10–15 treatments. The program included exercises to improve muscle strength and range of motion. Exercises were performed under the supervision of a physiotherapist, with a gradual increase in intensity and complexity. The aim of functional occupational therapy was to help patients perform everyday activities and improve their work abilities, as well as to improve movement coordination and functional independence. Analgesic electrotherapy procedures were applied to reduce pain and alleviate local inflammatory changes. Hydrokinesitherapy in thermomineral water was an integral part of the rehabilitation program (carbonic hyperthermal water—calcium, magnesium, hydrogen carbonate, sulfate, and carbon dioxide; temperature of 40–42 °C, 20 min), conducted daily over 21 days. All therapeutic modalities were applied over the same time period and with the same number of treatments for all patients, ensuring uniformity of the intervention for reliable monitoring and analysis of treatment outcomes. All therapies were conducted under the supervision of physiotherapists, who oversaw kinesiotherapy, electrotherapy procedures, and hydrotherapy sessions (including galvanic baths and thermomineral water therapy), while occupational therapy sessions were monitored and guided by certified occupational therapists.

This pragmatic multimodal protocol was intended to improve pain, movement, and daily function; it was not designed as a mechanism-specific treatment for central sensitization. Consequently, before–after changes cannot demonstrate that the protocol modified central sensitization pathways.

### 2.5. Sample Size Calculation

Sample size was determined a priori in G*Power 3.1 for a two-sided bivariate correlation with expected r = 0.30, alpha = 0.05, and 80% power, yielding a minimum of 84 participants. One hundred participants were enrolled and 97 completed the study. This calculation supports the prespecified correlation analysis only; it was not a power calculation for the multivariable regression models or paired before–after comparisons.

### 2.6. Bias

Consecutive eligible patients were invited to reduce selection bias. Information bias remains possible because the CSI, VAS, FACIT-Fatigue, BDI-II, and HAQ are patient-reported measures or include subjective components. The adjusted models included DAS28-CRP, ESR, and log-transformed IL-6 to represent complementary clinical and laboratory aspects of inflammatory activity. These covariates do not remove residual confounding, particularly when inflammatory-marker values have a restricted range.

### 2.7. Statistical Analysis

Continuous variables were summarized as means with standard deviations when approximately normally distributed and as medians (interquartile range) when skewed. Distribution normality was assessed using descriptive statistics, the Kolmogorov–Smirnov test, histograms, boxplots, and Q-Q plots. Baseline assessments were performed at admission (day 1). The before–after analyses were conducted using measurements obtained at admission and discharge (day 21) for VAS, HAQ, FACIT-Fatigue, CSI, ESR, CRP, IL-6, DAS28-ESR, and DAS28-CRP. BDI-II was included only in baseline analyses; no BDI-II change score or longitudinal inferential test is reported. Pearson correlation was used for approximately normal continuous variables and Spearman correlation for non-normal variables. Independent-group comparisons were conducted using the independent-samples *t* test or Mann–Whitney U test as indicated. Paired-samples *t* tests were used for approximately normally distributed before–after differences, and Wilcoxon signed-rank tests were used for skewed laboratory measures. Raw CRP and IL-6 values were summarized and correlated using nonparametric methods, while log-transformed values were used in linear regression. The regression analyses were interpreted as baseline cross-sectional models with VAS, HAQ, FACIT-Fatigue, and BDI-II as dependent variables and CSI score as the predictor of interest. Model 1 adjusted for DAS28-CRP; Model 2 additionally adjusted for ESR and log-transformed IL-6. All regression models were assessed for multicollinearity using the variance inflation factor, linearity, homoscedasticity, and residual normality using scatterplots, histograms, and Q-Q plots. One participant (1/97; 1.0%) had two missing values; all other participants had complete data, and no missing-value imputation was performed. Because the analyses were exploratory, no multiplicity adjustment was applied. All tests were two-sided, with *p* < 0.05. Analyses were performed in SPSS version 30.0 (IBM Corp., Armonk, NY, USA).

## 3. Results

### 3.1. Participant and Clinical Characteristics

One hundred women were enrolled and 97 completed the study and entered the final analysis. The demographic and clinical characteristics of the participants are summarized in Table 1. The mean age was 63.33 years (SD 9.18) and the mean BMI was 26.89 kg/m^2^ (SD 3.01). Forty-three participants (44.3%) smoked, 91 (93.8%) had at least one recorded comorbidity, 95 (97.9%) used a conventional synthetic DMARD, and 10 (10.3%) underwent biologic therapy. The median disease duration was 8 years. Radiographic stage was available for 97 participants; 92/97 (94.8%) were classified as stage I/II or higher. Baseline mean scores (SD) were 6.83 for VAS (1.31), 0.78 for HAQ (0.28), 28.40 for FACIT-Fatigue (8.76), 40.93 for CSI (14.84), 4.75 for DAS28-ESR (0.79), and 4.08 for DAS28-CRP (0.73). Fifty-seven participants (58.8%) had CSI scores of 40 or greater. At baseline, BDI-II categories among the 97 participants were minimal in 54 (55.7%), mild in 34 (35.1%), moderate in 9 (9.3%), and severe in 0 (0.0%).

**Table 1:** Demographic and clinical characteristics.

Characteristic	Value
Age, years	63.33 ± 9.18
Smoking	43 (44.3%)
BMI, kg/m^2^	26.89 ± 3.01
Comorbidities	91 (93.8%)
HTA	64 (66%)
HPT	25 (25.8%)
OP	24 (24.7%)
Gout	3 (3.1%)
CMP	8 (8.2%)
Bio Th	10 (10.3%)
csDMARD	95 (97.9%)
**Radiographic stage (n = 97)**	
I	5 (5.1%)
I/II	8 (8.3%)
II	47 (48.5%)
II/III	24 (24.7%)
III	9 (9.3%)
III/IV	4 (4.1%)

BMI: body mass index; HTA: arterial hypertension; HPT: hypothyroidism; OP: osteoporosis; CMP: cardiomyopathy; Bio Th: biologic therapy; csDMARD: conventional synthetic disease-modifying antirheumatic drug. Values are presented as n (%) or mean (standard deviation).

### 3.2. Correlation of Central Sensitization with Clinical Parameters

Higher CSI scores correlated with higher BDI-II, VAS, and DAS28-CRP scores and with lower FACIT-Fatigue scores, indicating greater fatigue. The DAS28-ESR correlation was borderline, and no statistically significant correlation was observed with CRP, IL-6, or disease duration. Participants with CSI scores ≥ 40 (n = 57) had higher mean DAS28-CRP than those with CSI scores below 40 (n = 40; 4.24 ± 0.69 vs. 3.85 ± 0.74; *p* = 0.010). The groups also differed in BDI-II, VAS, and FACIT-Fatigue, but not in age, BMI, disease duration, ESR, CRP, or IL-6 (Table 2).

**Table 2:** Associations of CSI score with age, BMI, laboratory and disease-activity parameters, and patient-reported outcomes.

Parameter	CSI Score, r (p)	CSI Below 40 (n = 40)	CSI ≥ 40 (n = 57)	*p* Value
Age, years	−0.164 (0.109) p	64.8 ± 8.5	62.3 ± 9.5	0.173 ^a^
BMI, kg/m^2^	0.009 (0.931) p	26.6 ± 3.1	27.1 ± 2.9	0.476 ^a^
Disease duration, years	0.042 (0.685) s	9 (9.75)	8 (9)	0.971 ^b^
BDI-II	0.394 (<0.001) p	8.3 ± 5.7	13.7 ± 5.5	<0.001 ^a^
ESR, mm/h	−0.072 (0.486) p	23.1 ± 13.1	22.9 ± 17.4	0.938 ^a^
CRP, mg/L	0.007 (0.948) s	3.67 (5.56)	3.4 (4.52)	0.956 ^b^
LogCRP	0.034 (0.741) p			
IL-6, pg/mL	0.013 (0.897) s	4.09 (8.37)	4.15 (6.06)	0.953 ^b^
LogIL-6	−0.070 (0.495) p			
VAS	0.380 (<0.001) p	6.44 ± 1.56	7.11 ± 1.03	0.012 ^a^
FACIT-Fatigue	−0.525 (<0.001) p	33.71 ± 8.99	24.77 ± 6.51	<0.001 ^a^
HAQ	0.036 (0.727) p	0.74 ± 0.27	0.80 ± 0.28	0.306 ^a^
DAS28-ESR	0.196 (0.055) p	4.58 ± 0.81	4.88 ± 0.75	0.064 ^a^
DAS28-CRP	0.253 (0.013) p	3.85 ± 0.74	4.24 ± 0.69	0.010 ^a^

BMI: body mass index; BDI-II: Beck Depression Inventory-II; ESR: erythrocyte sedimentation rate; CRP: C-reactive protein; log-transformed CRP; IL-6: interleukin-6; log-transformed IL-6; VAS: Visual Analog Scale; FACIT-Fatigue: Functional Assessment of Chronic Illness Therapy-Fatigue; HAQ: Health Assessment Questionnaire; DAS28: Disease Activity Score-28. p: Pearson correlation; s: Spearman correlation; ^a^: independent-samples *t* test; ^b^: Mann–Whitney U test. Raw CRP and IL-6 were analyzed nonparametrically; log-transformed CRP and IL-6 were used in regression.

### 3.3. Parameter Changes After Treatment

Across the three-week single-arm follow-up, the mean VAS score decreased by 2.53 points (SD 1.24), HAQ by 0.18 (SD 0.16), and CSI by 1.61 (SD 3.12), while the FACIT-Fatigue score increased by 2.34 points (SD 2.57). DAS28-ESR and DAS28-CRP also decreased, whereas ESR, CRP, and IL-6 did not change significantly. The mean FACIT-Fatigue change was below the 3–4-point minimally important difference [22], and the HAQ change was below the approximately 0.22-point benchmark commonly cited in RA [26]. These group-level benchmarks are interpretive and do not establish individual response. Individual responder rates were not available, and the absence of a control group precludes attribution of the changes to rehabilitation (Table 3).

**Table 3:** Parameter changes after treatment.

Parameter	Before	After	Δ	*p* Value
*Scores*				
VAS	6.83 ± 1.31	4.3 ± 1.29	−2.53 ± 1.24	<0.001 ^t^
HAQ	0.78 ± 0.28	0.6 ± 0.23	−0.18 ± 0.16	<0.001 ^t^
FACIT-Fatigue	28.40 ± 8.76	30.74 ± 8.32	2.34 ± 2.57	<0.001 ^t^
CSI score	40.93 ± 14.84	39.32 ± 14.31	−1.61 ± 3.12	<0.001 ^t^
*Laboratory*				
ESR, mm/h	20 (19.5)	20 (25)	0 (15)	0.086 ^w^
CRP, mg/L	3.55 (5.13)	2.51 (5.01)	−0.23 (3.75)	0.461 ^w^
IL-6, pg/mL	4.14 (6.71)	4.44 (7.96)	0.11 (3.86)	0.090 ^w^
*Disease activity*				
DAS28-ESR	4.75 ± 0.79	4.07 ± 0.83	−0.68 ± 0.65	<0.001 ^t^
DAS28-CRP	4.08 ± 0.73	3.28 ± 0.75	−0.79 ± 0.7	<0.001 ^t^

VAS: Visual Analog Scale; HAQ: Health Assessment Questionnaire; FACIT-Fatigue: Functional Assessment of Chronic Illness Therapy-Fatigue; CSI: Central Sensitization Inventory score; ESR: erythrocyte sedimentation rate; CRP: C-reactive protein; IL-6: interleukin-6; DAS28-ESR and DAS28-CRP: Disease Activity Score-28 calculated with ESR and CRP, respectively. Score values are shown as mean (standard deviation); laboratory values are shown as median (interquartile range). ^t^: paired-samples *t* test; ^w^: Wilcoxon signed-rank test.

### 3.4. Multivariable Regression Analysis

In baseline multivariable models, a higher CSI score was associated with a higher VAS pain score after adjustment for DAS28-CRP alone (unstandardized beta = 0.023; 95% CI 0.008 to 0.039; standardized beta = 0.265; *p* = 0.003) and after additional adjustment for ESR and log-transformed IL-6 (unstandardized beta = 0.024; 95% CI 0.008 to 0.040; standardized beta = 0.273; *p* = 0.003). A one-standard-deviation difference in CSI score (14.84 points) corresponds to approximately 0.36 VAS points, indicating a statistically significant but modest association. In Model 2, a higher CSI score was also associated with lower FACIT-Fatigue (unstandardized beta = −0.349; 95% CI −0.452 to −0.246; standardized beta = −0.590; *p* < 0.001) and higher BDI-II scores (unstandardized beta = 0.157; 95% CI 0.076 to 0.238; standardized beta = 0.375; *p* < 0.001), but not with HAQ (unstandardized beta = 0.001; 95% CI −0.004 to 0.004; *p* = 0.844). Model 2 total R^2^ values were 0.334 for VAS, 0.012 for HAQ, 0.369 for FACIT-Fatigue, and 0.208 for BDI-II; these values describe the complete models, not the unique variance explained by CSI (Table 4).

## 4. Discussion

Although pain in rheumatoid arthritis was traditionally considered exclusively a consequence of joint inflammation, more recent studies indicate the existence of numerous mechanisms that may be present even before the clinical manifestation of disease and that often do not correlate with the degree of inflammation or with the pharmacological therapy applied [27].

Inflammation, secondary osteoarthritis, and neurological mechanisms of central and peripheral sensitization play an important role in the onset and development of persistent residual pain in rheumatoid arthritis. Although DMARDs represent the therapeutic cornerstone, a significant number of patients do not achieve satisfactory pain control even with optimal anti-inflammatory therapy, which suggests that pain may also reflect mechanisms not fully captured by conventional measures of inflammatory disease activity [28,29].

Published estimates of elevated CSI scores in RA differ because cohorts vary in disease duration and activity and because studies use different instruments, thresholds, and case definitions [30]. Guler et al. reported 41% [31], Mesci et al. 48% [32], and Saitou et al. reported 7.5% at CSI ≥ 40 [33]. In the present cohort, 57/97 women (58.8%) had CSI scores ≥ 40. This threshold identifies elevated symptom burden but does not establish a neurophysiological diagnosis of central sensitization [20].

In this study, women with CSI scores ≥ 40 had higher BDI-II values than those with lower CSI scores. The baseline multivariable models also showed associations of higher CSI score with greater pain, more fatigue (lower FACIT-Fatigue), and more depressive symptoms, but not with HAQ. These are cross-sectional associations and should not be interpreted as evidence that CSI-defined symptom burden caused depression, fatigue, or pain. The adjusted pain coefficient was modest, and the reported R^2^ values describe the complete models rather than the unique contribution of CSI. During the three-week follow-up, VAS, FACIT-Fatigue, HAQ, CSI, DAS28-ESR, and DAS28-CRP improved, whereas ESR, CRP, and IL-6 did not change significantly. Because BDI-II is reported only in baseline analyses, this study does not evaluate change in depressive symptoms over 21 days.

Because these baseline associations are cross-sectional, their direction and causal meaning cannot be determined. Longer controlled studies measuring both variables repeatedly are needed.

Associations of CSI with VAS, FACIT-Fatigue, and BDI-II should be interpreted in the context of overlapping self-reported somatic and psychological symptoms. Hypothyroidism, osteoporosis, depressive symptoms, and other comorbidities may contribute to pain or fatigue and reduce the specificity of CSI-based classification. Although the adjusted fatigue and depression associations remained statistically significant in the reported models, this symptom overlap, the potentially bidirectional relationship between pain and depression [34], and residual confounding limit mechanistic interpretation.

The observed before–after changes are associations over time in an uncontrolled inpatient cohort. Regression to the mean, natural symptom fluctuation, improved physical function or muscle tension, expectancy effects, and nonspecific effects of a three-week inpatient stay are alternative explanations. The small CSI change, despite a larger VAS change, does not support a claim that the program substantially modified central sensitization mechanisms.

The reduction in CSI score was statistically significant but small (mean change −1.61 points) relative to the larger decrease in VAS score (mean change −2.53 points). This pattern does not demonstrate that the rehabilitation program modified central sensitization mechanisms. Because only baseline BDI-II results are reported, no conclusion can be drawn about short-term change in depressive symptoms.

Persistent pain is common even when inflammatory activity is low. Lee et al. reported persistent pain in DAS28 remission [35], and Vergne-Salle et al. described a substantial pain burden associated with psychological and disease-activity factors among patients receiving biologic DMARDs [36]. Perniola et al. likewise reported residual pain in clinical and ultrasound remission [37]. These studies support assessment of non-inflammatory contributors to pain but do not show that CSI alone identifies the underlying mechanism.

CSI score was positively correlated with DAS28-CRP score (r = 0.253, *p* = 0.013), and women with CSI scores ≥ 40 had higher mean DAS28-CRP score than those with scores below 40 (4.24 ± 0.69 vs. 3.85 ± 0.74; *p* = 0.010). In contrast, CSI was not significantly correlated with ESR, CRP, or IL-6. This divergence is consistent with a possible contribution from non-laboratory components of DAS28-CRP, including tender-joint count and patient global assessment. However, TJC28, SJC28, and PGA were not analyzed separately; the present data therefore cannot identify which component drove the association or establish that symptom burden was independent of inflammation. The restricted dispersion of inflammatory markers further reduces power to detect correlations, and systemic markers may miss low-grade or localized inflammation. Clinically, persistent pain should prompt assessment of both inflammatory and non-inflammatory contributors before escalating anti-inflammatory therapy, rather than assuming either mechanism from CSI or DAS28 alone [38].

The CSI is practical for screening but is not a gold-standard test of central sensitization. Quantitative sensory testing, including pressure-pain thresholds, temporal summation, and conditioned pain modulation, can provide complementary mechanistic information, although no single test constitutes a universally accepted diagnostic standard [21].

### Limitations

Several limitations of this study should be considered. First, baseline associations are cross-sectional, precluding conclusions about direction or causality; the before–after component is longitudinal but uncontrolled, and the absence of a control group limits interpretation of changes observed during rehabilitation. Second, although information on comorbidities and current pharmacological treatment was available, the influence of specific comorbid conditions and individual csDMARD regimens was not separately examined. Third, as noted above, the individual DAS28 components were not analyzed separately, which limits the ability to identify their specific contribution. Fourth, BDI-II is reported only as a baseline measure, so change in depressive symptoms during rehabilitation cannot be evaluated. Fifth, the three-week follow-up may be insufficient to detect a meaningful change in CSI. Sixth, the cohort included only women from a single tertiary rehabilitation center, so the findings cannot be generalized to men or to institutions with different patient profiles. Seventh, the CSI is a self-report screening instrument whose items overlap with depression, fatigue, hypothyroidism, osteoporosis, and other comorbidities; it does not replace objective neurophysiological assessment, and excluding patients receiving antidepressant therapy may limit generalizability to the broader RA population. Eighth, inflammatory markers had a restricted range, which limits inference from null correlations, and systemic markers may not capture low-grade or localized inflammation. Ninth, the sample size was calculated for the prespecified correlation analysis rather than for the multivariable models; complete adjustment-covariate coefficients and adjusted R^2^ values were not available in the supplied statistical output.

## 5. Conclusions

In this cohort of women with RA, elevated CSI scores were common and were associated with greater pain, fatigue, and depressive symptom burden. In adjusted baseline models, higher CSI score remained associated with pain, fatigue, and depressive symptoms, whereas no meaningful association was observed with functional disability (HAQ). The findings support using the CSI as one component of broader pain assessment, alongside evaluation of inflammatory activity, function, mood, and other pain mechanisms.

The data do not establish causality or rehabilitation efficacy. While the overall study employed a three-week prospective follow-up, the associations reported here were derived from baseline cross-sectional analyses, which limit the extent to which fatigue and depression can be attributed specifically to central sensitization, given their shared reliance on self-reported symptoms and potential overlap with other comorbidities. Future controlled studies including women and men, broader inflammatory activity, objective sensory testing, complete DAS28 component data, and longer follow-up are needed.

## Figures and Tables

**Table 4:** Baseline associations of CSI score with VAS, HAQ, FACIT-Fatigue, and BDI-II after adjustment for inflammatory measures.

	CSI Score	Beta (95% CI)	*p* Value	Std. Beta	Partial Corr.	R/R^2^
VAS	Model 1	0.023 (0.008; 0.039)	0.003	0.265	0.298	0.573/0.329
	Model 2	0.024 (0.008; 0.040)	0.003	0.273	0.305	0.578/0.334
HAQ	Model 1	0.001 (−0.004; 0.004)	0.867	0.018	0.017	0.106/0.011
	Model 2	0.001 (−0.004; 0.004)	0.844	0.021	0.021	0.109/0.012
FACIT-Fatigue	Model 1	−0.338 (−0.444; −0.233)	<0.001	−0.571	−0.553	0.553/0.306
	Model 2	−0.349 (−0.452; −0.246)	<0.001	−0.590	−0.579	0.608/0.369
BDI-II	Model 1	0.146 (0.066; 0.226)	<0.001	0.349	0.350	0.426/0.182
	Model 2	0.157 (0.076; 0.238)	<0.001	0.375	0.374	0.457/0.208

N = 97. CSI: Central Sensitization Inventory; VAS: Visual Analog Scale; HAQ: Health Assessment Questionnaire; FACIT-Fatigue: Functional Assessment of Chronic Illness Therapy-Fatigue; BDI-II: Beck Depression Inventory-II; ESR: erythrocyte sedimentation rate; LogIL-6: log-transformed interleukin-6. R and R^2^ refer to the complete model; adjusted R^2^ was not available. All values are baseline values. Model 1 adjusted for DAS28-CRP; Model 2 adjusted for DAS28-CRP, ESR, and LogIL-6. Table 4 reports the CSI coefficient from each model. Complete coefficients for the adjustment covariates and adjusted R^2^ values were not available in the supplied statistical output and therefore are not presented.

## Data Availability

The data associated with the paper are available from the corresponding author upon reasonable request.
